# Growth Hormone Cotreatment for Low-Prognosis Patients According to the POSEIDON Criteria

**DOI:** 10.3389/fendo.2021.790160

**Published:** 2021-12-02

**Authors:** Xueying Liu, Jingxiao Xu, Lixin Bi, Peihao Liu, Xue Jiao

**Affiliations:** ^1^ Center for Reproductive Medicine, Cheeloo College of Medicine, Shandong University, Jinan, China; ^2^ Key Laboratory of Reproductive Endocrinology of Ministry of Education, Shandong University, Jinan, China; ^3^ Shandong Key Laboratory of Reproductive Medicine, Jinan, China; ^4^ Shandong Provincial Clinical Research Center for Reproductive Health, Jinan, China; ^5^ National Research Center for Assisted Reproductive Technology and Reproductive Genetics, Shandong University, Jinan, China

**Keywords:** growth hormone, poor ovarian response, POSEIDON criteria, pregnancy outcome, *in vitro* fertilization

## Abstract

**Background:**

Poor ovarian response (POR) remains one of the most challenging conditions in assisted reproduction technology. Previous studies seemed to indicate that growth hormone (GH) was a potential solution for the dilemma of POR; however, the role GH played on the low-prognosis patients diagnosed and stratified by the POSEIDON criteria remains indistinct.

**Methods:**

This retrospective study was performed among women with POR according to the POSEIDON criteria who failed a previous *in vitro* fertilization (IVF)/intracytoplasmic sperm injection (ICSI) cycle, and the subsequent cycle was under GH cotreatment and conducted within 12 months. These participants were stratified into four groups according to the POSEIDON criteria. The comparison was implemented between the failed cycle and the cycle treated with GH. Generalized estimating equation (GEE) multivariate regression was applied for data analysis.

**Results:**

A total of 428 low-prognosis women were included in this study. GH supplementation improved the live birth rates (47.66%, 28.33%, 45.45%, and 24.07%; in groups 1, 2, 3, and 4, respectively) and the clinical pregnancy rates (OR 19.16, 95% CI 7.87–46.63, *p* < 0.001; OR 7.44, 95% CI 1.65–33.55, *p* = 0.009; OR 10.19, 95% CI 2.39–43.52, *p* = 0.002; OR 27.63, 95% CI 4.46–171.11, *p* < 0.001; in groups 1, 2, 3, and 4, respectively) in all four POSEIDON groups. The number of oocytes retrieved was significantly elevated in the subgroups with normal ovarian reserve (IRR 1.47, 95% CI 1.36–1.59, *p* < 0.001; IRR 1.31, 95% CI 1.15–1.49, *p* < 0.001; in groups 1 and 2, respectively). The number of day-3 good-quality embryos was significantly elevated in the subgroups with either normal ovarian reserve or aged young (IRR 2.13, 95% CI 1.78–2.56, *p* < 0.001; IRR 1.54, 95% CI 1.26–1.89, *p* < 0.001; IRR 1.47, 95% CI 1.10–1.98, *p* = 0.010; in groups 1, 2, and 3, respectively).

**Conclusion:**

Growth hormone cotreatment could ameliorate the pregnancy outcome for women with POR under the POSEIDON criteria who failed a previous IVF/ICSI cycle. The application of growth hormone for low-prognosis women who experienced a failed cycle might be considered and further studied.

## Introduction

Although it is well acknowledged that poor ovarian response (POR) is one of the most arduous challenges in the assisted reproductive technology (ART), its definition used to be inconsistent among studies (1). In 2011, the Bologna criteria were presented by the European Society of Human Reproduction and Embryology (ESHRE) to homogenize the POR population (2). However, subsequent studies revealed that the group of POR patients defined by the Bologna criteria was clinically heterogeneous, which was primarily due to the ignorance of the effect of age on oocyte quality (3–5).

To settle this issue, the Patient-Oriented Strategies Encompassing Individualized Oocyte Number (POSEIDON) criteria for POR were proposed in 2016 (6), and they stratified all patients with POR into four groups according to maternal age, ovarian biomarkers, and the ovarian response to regular ovarian stimulation protocols. The POSEIDON criteria are viewed to be preferable than the Bologna criteria since the oocyte quality is taken into consideration and women in each subgroup shared more homogeneous characteristics, which could eventually benefit the individualized management in the clinical practice.

Growth hormone (GH) is a 191-amino-acid polypeptide hormone secreted from the pituitary gland (7) and is initially applied for conditions leading to GH deficiency. GH is reported to be capable of enhancing the functional mitochondria in oocytes (8), improving the proliferation and differentiation of granulosa cells (9, 10) and increasing the endometrial blood perfusion as well (11). In seeking an effective approach to tackle the challenges of POR, GH has been explored in many related studies (8, 12–14) and utilized clinically for patients with POR during the ovarian stimulation for *in vitro* fertilization (IVF)/intracytoplasmic sperm injection (ICSI) as well. Thus, it is crucial to ascertain the validity and specific effects of GH supplementation on the management of patients with POR. A recent systematic review and meta-analysis by Cozzolino et al. assessed the effectiveness of GH supplementation for POR patients and revealed that GH could increase the number of oocytes retrieved, the number of MII oocytes, the number of embryos available to transfer, and the clinical pregnancy rate (15). Yet another recent review among women with Bologna-criteria POR found that although GH could reduce the gonadotrophin (Gn) dosage required it did not improve the clinical pregnancy rate in IVF cycles, so the authors speculated that the response to GH might differ in subgroups of POR patients (16).

Many of the previous studies seemed to be indicating that GH was a promising solution for the dilemma of POR; however, they included patients with POR under Bologna criteria, or even published before the definition of the Bologna criteria. The role GH played on the low-prognosis patients diagnosed and stratified by the POSEIDON criteria is still unclear. The efficacy of GH on POR patients in specific subgroups with different ages and ovarian reserve is yet to be confirmed.

This was a retrospective study aiming to investigate the effectiveness of GH addition in the IVF/ICSI outcomes for poor responders defined by the POSEIDON criteria. These findings will offer valuable clues for the clinical application of GH for different POR subpopulation, especially for those patients uncovered by the Bologna criteria.

## Materials and Methods

### Participants

This retrospective study was performed among women diagnosed as POR according to the POSEIDON criteria who failed a previous IVF/ICSI cycle, and the subsequent cycle was under GH cotreatment and conducted within 12 months in the Center for Reproductive Medicine, Shandong University, from March 2018 to December 2020. The comparison was implemented between the failed cycle (non-GH cycle) and the cycle treated with GH (GH cycle).

The participants were divided into four groups according to the POSEIDON classification (6). POSEIDON group 1 consists of patients <35 years old and anti-Müllerian hormone (AMH) ≥1.2 ng/ml and an unexpected poor (<3 oocytes retrieved) or suboptimal (four to nine oocytes retrieved) response. POSEIDON group 2 consists of patients ≥35 years old with AMH ≥ 1.2 ng/ml and an unexpected poor or suboptimal ovarian response. POSEIDON group 3 consists of patients <35 years old and AMH <1.2 ng/ml; POSEIDON group 4: patients ≥ 35 years and AMH < 1.2 ng/ml. We chose AMH concentration to divide the four groups because AMH was considered to be more accurate and robust than antral follicle count (AFC) (17, 18).

Exclusion criteria were as follows. (1) Different fertilization methods (IVF vs. ICSI) applied in the previous and the subsequent cycle. (2) The protocol of controlled ovarian hyperstimulation (COH) was neither gonadotropin-releasing hormone (GnRH) antagonist nor agonist protocol. (3) Either one of the couples had chromosomal abnormalities. The study was approved by the Institutional Review Board (IRB) of the Center for Reproductive Medicine, Shandong University. All the participants included in this study had signed written informed consent.

### Clinical Management

COH protocols including GnRH antagonist protocols and GnRH agonist ultra-long/long/short protocols were applied individually according to patients’ conditions. In the GnRH antagonist protocol, follicle-stimulating hormone (FSH) was administered on day 3 of the menstrual cycle. When at least one follicle was 12 mm or more, the GnRH antagonist (ganirelix, MSD Organon, Oss, Netherlands) at a dose of 0.25 mg daily was started and continued until the day of human chorionic gonadotropin (hCG) triggering. In the GnRH agonist ultra-long/long protocols, the patients received a long-acting GnRH agonist (leuprolide acetate, 3.75 mg) at previous cycle days 1–3 or 0.1 mg triptorelin daily injection from previous cycle day 21 for 14 days, followed by ovulation stimulation with Gn 28–35 or 14 days later. In the GnRH agonist short protocol, patients were administered 0.05/0.1 mg triptorelin daily injection on day 3 of the cycle, then administered Gn starting from days 4 or 5 of the cycle. In all treatment protocols, the ovaries were stimulated with FSH (Gonal-F; Merck Serono, Geneva, Switzerland; or Puregon; MSD Organon, Oss, Netherlands; or urofollitropin for injection; Livzon Pharmaceutical Group, Zhuhai, China) or human menopausal gonadotropin (menotropins for injection; Livzon Pharmaceutical Group, Zhuhai, China).

The monitoring of ovarian response and adjustment of the dose of Gn during COH were assessed by ultrasound examination and hormone concentrations. Ovulation was induced with hCG when at least one follicle was 18 mm or greater in mean diameter. Oocyte retrieval was completed 34–36 h following hCG administration by transvaginal ultrasound guidance.

GH-treated patients received 1–2 IU/day of GH (Saizen; Changchun GeneScience, Changchun, China; or GenHeal; Shanghai United Cell Biotechnology, Shanghai, China), beginning in the previous menstrual cycle (in the follicular phase or luteal phase, 1–5 weeks before Gn) and maintained until the day of triggering.

### Embryo Assessment

Day-3 embryos were graded based on the number and size of blastomere and the degree of fragmentation (19). Day-5/6 embryos were graded using the Gardner scoring system (20) with respect to the inner cell mass and trophectoderm. On day 3 or day 5 of the embryo culture, up to two embryos were selected and transferred. Supernumerary blastocysts suitable for transfer were cryopreserved.

### Outcome Measures

Day-3 good-quality embryos referred to the embryo reached the 7–10-cell stage and ≤20% fragmentation on day 3. Embryos available to transfer denoted the number of embryos transferred plus the number of supernumerary frozen blastocysts. Chemical pregnancy was defined as serum β-hCG level >10 IU/l 14 days after the embryo transfer. Clinical pregnancy was defined as the presence of a gestational sac by transvaginal ultrasound evaluation 5 weeks after embryo transfer. Live birth was defined as the live delivery of at least one fetus after 28 weeks of gestation.

### Statistical Analysis

Data were summarized using the mean and standard deviation for quantitative variables and the number and percentage for qualitative variables. Quantitative variables were compared using paired t-test. Qualitative variables were analyzed using the chi-square test, with Fisher’s exact test for expected frequencies less than five, as appropriate. Generalized estimating equations (GEE) were applied to assess the effect of GH supplementation in the numbers of oocytes retrieved, day-3 good-quality embryos and embryos available to transfer, and rates of chemical pregnancy and clinical pregnancy, after accounting for several confounders. The candidate confounders were age, body mass index (BMI), AMH, FSH, AFC, COH protocols, Gn duration, and total Gn dose. Results are presented with incidence rate ratios (IRRs) or odds ratios (ORs) and 95% confidence intervals (CIs). Statistical significance was assumed when *p* < 0.05. All analyses performed using STATA 15.1.

## Results

Our study included 428 eligible patients who were assigned to four groups based on the POSEIDON criteria: 151 women in the POSEIDON group 1, 101 women in the POSEIDON group 2, 71 women in the POSEIDON group 3, and 105 women in the POSEIDON group 4. Baseline characteristics and detailed usage of GH of all patients are summarized in **Table 1**. The cycle characteristics are shown in **Table 2**. Patients used more Gn dose (group 1: 2304.64 ± 1116.89 *vs*. 2006.62 ± 893.69, *p* = 0.001; group 2: 2667.95 ± 940.62 *vs*. 2153.84 ± 919.26, *p <* 0.001; group 3: 2893.49 ± 1196.39 *vs*. 2397.75 ± 1351.87, *p* = 0.017; group 4: 3268.93 ± 1831.57 *vs*. 2478.21 ± 1163.91, *p* < 0.001) and underwent longer duration of Gn treatments (group 2: 9.91 ± 1.98 *vs*. 9.43 ± 2.10, *p* = 0.037; group 4: 10.45 ± 3.30 *vs*. 9.24 ± 2.20, *p* = 0.001) in the GH-treated cycles than patients in non-GH cycles. COH protocols were different between the GH-treated cycles and the non-GH cycles in the four POSEIDON groups (*p* < 0.001).

**Table 1:** Baseline characteristics of participants in the POSEIDON groups.

	POSEIDON	POSEIDON	POSEIDON	POSEIDON
	group 1 (n = 151)	group 2 (n = 101)	group 3 (n = 71)	group 4 (n = 105)
Age (years)	30.15 ± 2.50	38.80 ± 2.86	30.63 ± 2.77	38.77 ± 2.89
BMI (kg/m^2^)	23.44 ± 3.52	24.26 ± 2.94	23.54 ± 3.30	24.13 ± 2.74
Infertility duration (years)	3.39 ± 2.09	5.14 ± 4.46	3.54 ± 2.59	4.30 ± 3.52
Cause of infertility				
Female factor	109 (72.19)	90 (89.11)	55 (77.46)	85 (80.95)
Male factor	27 (17.88)	7 (6.93)	9 (12.68)	12 (11.43)
Combined factors	12 (7.95)	3 (2.97)	5 (7.04)	8 (7.62)
Unexplained infertility	3 (1.99)	1 (0.99)	2 (2.82)	0 (0)
Baseline sex hormone				
FSH (IU/L)	7.02 ± 1.71	7.14 ± 1.98	8.31 ± 3.44	8.98 ± 3.52
LH (IU/L)	5.32 ± 3.05	4.38 ± 2.05	4.18 ± 1.93	4.86 ± 3.40
Estradiol (pg/mL)	37.77 ± 14.73	41.53 ± 16.74	42.06 ± 17.52	43.56 ± 17.31
AMH (ng/mL)	3.22 ± 2.46	2.29 ± 1.70	0.77 ± 0.31	0.65 ± 0.33
AFC	11.87 ± 5.32	9.50 ± 4.35	6.86 ± 2.54	6.10 ± 2.38
GH starting time				
Luteal phase of the previous cycle	93 (61.59)	57 (56.44)	32 (45.07)	57 (54.29)
Follicular phase of the previous cycle	58 (38.41)	44 (43.56)	39 (54.93)	48 (45.71)
GH dosage per day				
2 IU	129 (85.43)	91 (90.10)	62 (87.32)	97 (92.38)
1.5 IU	8 (5.30)	2 (1.98)	3 (4.23)	2 (1.90)
1 IU	14 (9.27)	8 (7.92)	6 (8.45)	6 (5.71)

All values presented as mean ± SD or n (%).

BMI, body mass index; FSH, follicle-stimulating hormone; LH, luteinizing hormone; AMH, anti-Müllerian hormone; AFC, antral follicle count; GH, growth hormone.

**Table 2:** Parameters during the ovarian stimulation in the POSEIDON groups.

	POSEIDON group 1 (n = 151)			POSEIDON group 2 (n = 101)			POSEIDON group 3 (n = 71)			POSEIDON group 4 (n = 105)		
	GH	Non-GH	*p*-value	GH	Non-GH	*p*-value	GH	Non-GH	*p*-value	GH	Non-GH	*p*-value
COH protocol			<0.001*			<0.001*			<0.001*			<0.001*
GnRH antagonist	33 (21.85)	50 (33.11)	0.028*	29 (28.71)	21 (20.79)	0.192	21 (29.58)	20 (28.17)	0.853	34 (32.38)	31 (29.52)	0.654
GnRH agonist long	71 (47.02)	49 (32.45)	0.010*	41 (40.59)	15 (14.85)	<0.001*	21 (29.58)	12 (16.90)	0.074	24 (22.86)	7 (6.67)	0.001*
GnRH agonist ultra-long	26 (17.22)	7 (4.64)	<0.001*	15 (14.85)	4 (3.96)	0.008*	19 (26.76)	2 (2.82)	<0.001*	19 (18.10)	4 (3.81)	0.001*
GnRH agonist short	21 (13.91)	45 (29.80)	0.001*	16 (15.84)	61 (60.40)	<0.001*	10 (14.08)	37 (52.11)	<0.001*	28 (26.67)	63 (60.00)	<0.001*
Duration of Gn (days)	9.52 ± 2.15	9.65 ± 2.16	0.536	9.91 ± 1.98	9.43 ± 2.10	0.037*	9.85 ± 2.09	9.55 ± 2.71	0.458	10.45 ± 3.30	9.24 ± 2.20	0.001*
Total Gn dose (IU)	2304.64 ± 1116.89	2006.62 ± 893.69	0.001*	2667.95 ± 940.62	2153.84 ± 919.26	<0.001*	2893.49 ± 1196.39	2397.75 ± 1351.87	0.017*	3268.93 ± 1831.57	2478.21 ± 1163.91	<0.001*
E_2_ levels on hCG day (pg/mL)	2693.01 ± 1566.16	2229.32 ± 1032.86	<0.001*	2365.51 ± 1184.93	2293.50 ± 1050.46	0.586	1822.62 ± 1181.90	1742.50 ± 792.48	0.466	1604.78 ± 956.03	1484.89 ± 752.35	0.180
Endometrial thickness on hCG day (mm)	10.52 ± 2.01	10.72 ± 2.07	0.129	10.43 ± 2.21	9.99 ± 1.96	0.001*	10.51 ± 1.91	10.54 ± 1.84	0.860	10.02 ± 2.41	9.64 ± 2.40	0.013*

All values presented as mean ± SD or n (%).

GH, growth hormone; COH, controlled ovarian hyperstimulation; GnRH, gonadotropin-releasing hormone; Gn, gonadotropin; E_2_, estradiol; hCG, human chorionic gonadotropin.

*indicates statistical significances of p < 0.05.

The clinical outcomes are shown in **Table 3**. In the POSEIDON groups 1, 2, and 3, the numbers of oocytes retrieved (group 1: 8.62 ± 4.30 *vs*. 5.62 ± 2.40, *p* < 0.001; group 2: 6.86 ± 3.52 *vs*. 5.29 ± 2.04, *p* < 0.001; group 3: 5.27 ± 2.98 *vs*. 4.45 ± 2.37, *p* = 0.025), day-3 good-quality embryos (group 1: 2.31 ± 2.22 *vs*. 1.13 ± 1.28, *p* < 0.001; group 2: 2.61 ± 2.23 *vs*. 1.76 ± 1.52, *p* < 0.001; group 3:1.63 ± 1.65 *vs*. 0.96 ± 1.22, *p* = 0.002), and embryos available to transfer (group 1: 2.54 ± 1.87 *vs*. 1.15 ± 0.91, *p* < 0.001; group 2: 2.12 ± 1.63 *vs*. 1.44 ± 1.14, *p* < 0.001; group 3: 1.86 ± 1.53 *vs*. 0.94 ± 0.97, *p* < 0.001) were significantly increased in the GH-treated cycle compared to the non-GH cycle. In the POSEIDON group 4, the number of embryos available to transfer (1.32 ± 1.16 *vs*. 0.96 ± 0.90, *p* = 0.004) was significantly increased in the GH-treated cycle than in the non-GH cycle. No statistically significant differences existed in the numbers of oocytes retrieved and day-3 good-quality embryos (*p* > 0.05).

**Table 3:** Comparison of the clinical outcomes in the POSEIDON groups.

	POSEIDON group 1 (n = 151)			POSEIDON group 2 (n = 101)			POSEIDON group 3 (n = 71)			POSEIDON group 4 (n = 105)		
	GH	Non-GH	*p*-value	GH	Non-GH	*p*-value	GH	Non-GH	*p*-value	GH	Non-GH	*p*-value
No. of oocytes retrieved	8.62 ± 4.30	5.62 ± 2.40	<0.001*	6.86 ± 3.52	5.29 ± 2.04	<0.001*	5.27 ± 2.98	4.45 ± 2.37	0.025*	4.14 ± 2.95	3.73 ± 2.45	0.213
No. of two pronuclei	5.30 ± 3.08	3.22 ± 1.77	<0.001*	4.47 ± 2.84	3.43 ± 1.85	0.001*	3.28 ± 2.45	2.77 ± 1.88	0.057	2.63 ± 2.22	2.63 ± 1.76	1.000
No. of day-3 good-quality embryos	2.31 ± 2.22	1.13 ± 1.28	<0.001*	2.61 ± 2.23	1.76 ± 1.52	<0.001*	1.63 ± 1.65	0.96 ± 1.22	0.002*	1.25 ± 1.39	1.16 ± 1.11	0.594
No. of embryos available to transfer	2.54 ± 1.87	1.15 ± 0.91	<0.001*	2.12 ± 1.63	1.44 ± 1.14	<0.001*	1.86 ± 1.53	0.94 ± 0.97	<0.001*	1.32 ± 1.16	0.96 ± 0.90	0.004*
Stage of embryo transferred			0.507			0.797			0.192			0.334
Cleavage-stage embryo	85 (79.44)	71 (75.53)		48 (80.00)	39 (78.00)		39 (88.64)	24 (77.42)		42 (77.78)	34 (69.39)	
Blastocyst	22 (20.56)	23 (24.47)		12 (20.00)	11 (22.00)		5 (11.36)	7 (22.58)		12 (22.22)	15 (30.61)	
No. of embryos transferred			0.044*			0.801			0.407			0.096
One embryo transferred	32 (29.91)	41 (43.62)		23 (38.33)	18 (36.00)		13 (29.55)	12 (38.71)		22 (40.74)	28 (57.14)	
Two embryos transferred	75 (70.09)	53 (56.38)		37 (61.67)	32 (64.00)		31 (70.45)	19 (61.29)		32 (59.26)	21 (42.86)	
Chemical pregnancy rate/ET	62/107 (57.94)	25/94 (26.60)	<0.001*	27/60 (45.00)	14/50 (28.00)	0.066	25/44 (56.82)	6/31 (19.35)	0.001*	25/54 (46.30)	6/49 (12.24)	<0.001*
Clinical pregnancy rate/ET	58/107 (54.21)	7/94 (7.45)	<0.001*	21/60 (35.00)	5/50 (10.00)	0.002*	21/44 (47.73)	3/31 (9.68)	0.001*	20/54 (37.04)	2/49 (4.08)	<0.001*
Clinical pregnancy loss rate/CP	7/58 (12.07)	7/7 (100.00)	<0.001*	4/21 (19.05)	5/5 (100.00)	0.002*	1/21 (4.76)	3/3 (100.00)	0.002*	7/20 (35.00)	2/2 (100.00)	0.049*
Live birth rate/ET	51/107 (47.66)	0	<0.001*	17/60 (28.33)	0	<0.001*	20/44 (45.45)	0	<0.001*	13/54 (24.07)	0	<0.001*

All values presented as mean ± SD or n (%).

GH, growth hormone; ET, embryo transfer; CP, clinical pregnancy.

In bold: The values associated with GH.

*indicates statistical significances of p < 0.05.

The results of GEE regression analysis of factors related to the numbers of oocytes retrieved, day-3 good-quality embryos, and embryos available to transfer are presented in **Tables 4**–**6**. GH supplementation was a significant predictor of the number of oocytes retrieved (group 1: IRR 1.47, 95% CI 1.36–1.59, *p* < 0.001; group 2: IRR 1.31, 95% CI 1.15–1.49, *p* < 0.001; **Table 4**) of patients in POSEIDON group 1 and group 2 and the number of day-3 good-quality embryos (group 1: IRR 2.13, 95% CI 1.78–2.56, *p* < 0.001; group 2: IRR 1.54, 95% CI 1.26–1.89, *p* < 0.001; group 3: IRR 1.47, 95% CI 1.10–1.98, *p* = 0.010; **Table 5**) of patients in POSEIDON groups 1, 2, and 3, and it also was a significant predictor of the numbers of embryos available to transfer (group 1: IRR 2.33, 95% CI 1.96–2.77, *p* < 0.001; group 2: IRR 1.57, 95% CI 1.26–1.95, *p* < 0.001; group 3: IRR 2.06, 95% CI 1.51–2.82, *p* < 0.001; group 4: IRR 1.31, 95% CI 1.02–1.69, *p* = 0.033; **Table 6**) of patients in the four POSEIDON groups after conducting GEE multivariate regression to adjust for relevant confounders.

**Table 4:** GEE regression analysis for the number of oocytes retrieved.

Independent variable	POSEIDON group 1			POSEIDON group 2			POSEIDON group 3			POSEIDON group 4		
	IRR	95% CI	*p*-value	IRR	95% CI	*p*-value	IRR	95% CI	*p*-value	IRR	95% CI	*p*-value
GH supplementation												
Non-GH	_	_		_	_		_	_		_	_	
GH	1.47	1.36–1.59	<0.001*	1.31	1.15–1.49	<0.001*	1.17	1.00–1.36	0.054	1.04	0.89–1.20	0.637
Age	1.00	0.99–1.02	0.624	1.00	0.98–1.02	0.838	1.01	0.98–1.05	0.355	1.00	0.98–1.03	0.885
BMI	0.98	0.97–1.00	0.033*	0.99	0.97–1.01	0.231	1.01	0.99–1.01	0.458	1.00	0.97–1.02	0.766
AMH	1.04	1.02–1.07	0.001*	0.96	0.92–1.00	0.062	1.68	1.24–2.28	0.001*	1.60	1.24–2.05	<0.001*
FSH	0.99	0.96–1.03	0.741	0.95	0.92–0.99	0.005*	0.98	0.95–1.01	0.124	0.97	0.95–0.99	0.013*
AFC	1.01	1.00–1.02	0.075	1.03	1.01–1.05	0.001*	1.02	0.98–1.06	0.352	1.01	0.98–1.05	0.381
COH protocol												
GnRH antagonist	_	_		_	_		_	_		_	_	
GnRH agonist long	1.20	1.08–1.32	<0.001*	1.14	0.97–1.33	0.114	1.12	0.92–1.37	0.261	1.31	1.06–1.62	0.012*
GnRH agonist ultra-long	1.05	0.91–1.22	0.490	1.31	1.06–1.62	0.014*	0.84	0.64–1.10	0.209	1.16	0.89–1.51	0.268
GnRH agonist short	1.09	0.96–1.24	0.190	1.23	1.05–1.45	0.010*	0.91	0.75–1.11	0.353	1.09	0.91–1.29	0.355
Duration of Gn	1.00	0.97–1.02	0.740	1.02	0.98–1.07	0.292	1.05	1.00–1.10	0.033*	1.04	0.99–1.09	0.121
Gn dose (per 100 IU)	1.01	1.00–1.01	0.006*	1.00	0.99–1.01	0.777	1.00	0.99–1.01	0.735	1.00	1.00–1.00	0.574

GH, growth hormone; BMI, body mass index; AMH, anti-Müllerian hormone; FSH, follicle-stimulating hormone; AFC, antral follicle count; COH, controlled ovarian hyperstimulation; GnRH, gonadotropin-releasing hormone; Gn, gonadotropin.

In bold: The values associated with GH.

*indicates statistical significances of p < 0.05.

**Table 5:** GEE regression analysis for the number of day-3 good-quality embryos.

Independent variable	POSEIDON group 1			POSEIDON group 2			POSEIDON group 3			POSEIDON group 4		
	IRR	95% CI	*p*-value	IRR	95% CI	*p*-value	IRR	95% CI	*p*-value	IRR	95% CI	*p*-value
GH supplementation												
Non-GH	_	_		_	_		_	_		_	_	
GH	2.13	1.78–2.56	<0.001*	1.54	1.26–1.89	<0.001*	1.47	1.10–1.98	0.010*	1.00	0.78–1.30	0.971
Age	1.02	0.98–1.06	0.271	1.02	0.98–1.06	0.356	1.00	0.94–1.06	0.890	0.98	0.93–1.03	0.354
BMI	0.98	0.95–1.00	0.093	0.96	0.93–1.00	0.042*	1.02	0.97–1.08	0.377	0.99	0.94–1.05	0.845
AMH	1.06	1.01–1.11	0.014*	0.92	0.84–1.00	0.043*	2.02	1.07–3.83	0.030*	0.88	0.55–1.39	0.573
FSH	0.96	0.91–1.02	0.222	0.94	0.89–0.99	0.031*	1.03	0.98–1.09	0.237	0.99	0.95–1.04	0.756
AFC	0.99	0.97–1.01	0.379	1.04	1.01–1.07	0.012*	1.04	0.97–1.13	0.270	0.99	0.93–1.06	0.792
COH protocol												
GnRH antagonist	_	_		_	_		_	_		_	_	
GnRH agonist long	1.25	1.01–1.55	0.037*	1.24	0.96–1.60	0.099	1.35	0.94–1.96	0.108	1.74	1.20–2.52	0.004*
GnRH agonist ultra-long	0.57	0.39–0.83	0.003*	1.05	0.73–1.52	0.784	1.28	0.80–2.05	0.311	1.53	0.95–2.46	0.081
GnRH agonist short	1.31	1.01–1.70	0.038*	1.33	1.02–1.74	0.038*	0.76	0.51–1.13	0.178	1.09	0.80–1.48	0.600
Duration of Gn	0.99	0.93–1.04	0.628	1.05	0.98–1.13	0.158	1.02	0.93–1.12	0.642	1.07	0.98–1.17	0.144
Gn dose (per 100 IU)	1.01	0.99–1.02	0.272	1.00	0.99–1.01	0.994	0.99	0.97–1.01	0.219	1.00	1.00–1.00	0.024*

GH, growth hormone; BMI, body mass index; AMH, anti-Müllerian hormone; FSH, follicle-stimulating hormone; AFC, antral follicle count; COH, controlled ovarian hyperstimulation; GnRH, gonadotropin-releasing hormone; Gn, gonadotropin.

In bold: The values associated with GH.

*indicates statistical significances of p < 0.05.

**Table 6:** GEE regression analysis for the number of embryos available to transfer.

Independent variable	POSEIDON group 1			POSEIDON group 2			POSEIDON group 3			POSEIDON group 4		
	IRR	95% CI	*p*-value	IRR	95% CI	*p*-value	IRR	95% CI	*p*-value	IRR	95% CI	*p*-value
GH supplementation												
Non-GH	_	_		_	_		_	_		_	_	
GH	2.33	1.96–2.77	<0.001*	1.57	1.26–1.95	<0.001*	2.06	1.51–2.82	<0.001*	1.31	1.02–1.69	0.033*
Age	1.00	0.96–1.04	0.993	0.98	0.94–1.02	0.369	1.02	0.96–1.08	0.539	0.99	0.95–1.04	0.796
BMI	0.98	0.95–1.01	0.173	0.97	0.93–1.01	0.135	1.05	1.00–1.10	0.037*	0.98	0.93–1.04	0.500
AMH	1.06	1.01–1.11	0.011*	0.94	0.86–1.03	0.213	2.29	1.27–4.13	0.006*	0.97	0.59–1.59	0.899
FSH	1.01	0.95–1.07	0.771	0.95	0.89–1.02	0.157	0.99	0.93–1.04	0.599	0.99	0.95–1.03	0.661
AFC	0.98	0.96–1.00	0.118	1.02	0.99–1.06	0.178	1.01	0.94–1.08	0.806	1.02	0.95–1.08	0.654
COH protocol												
GnRH antagonist	_	_		_	_		_	_		_	_	
GnRH agonist long	1.15	0.94–1.41	0.166	1.23	0.94–1.61	0.130	1.01	0.69–1.48	0.955	1.46	1.02–2.11	0.041*
GnRH agonist ultra-long	0.91	0.66–1.24	0.551	1.01	0.67–1.51	0.971	0.88	0.55–1.41	0.597	1.30	0.82–2.07	0.260
GnRH agonist short	1.24	0.97–1.60	0.090	1.18	0.88–1.58	0.261	0.95	0.64–1.40	0.799	1.02	0.75–1.39	0.915
Duration of Gn	1.04	0.98–1.10	0.169	1.05	0.97–1.13	0.235	1.08	0.98–1.17	0.108	1.05	0.96–1.15	0.284
Gn dose (per 100 IU)	0.99	0.98–1.00	0.150	0.99	0.97–1.00	0.130	0.99	0.97–1.00	0.139	1.00	1.00–1.00	0.083

GH, growth hormone; BMI, body mass index; AMH, anti-Müllerian hormone; FSH, follicle-stimulating hormone; AFC, antral follicle count; COH, controlled ovarian hyperstimulation; GnRH, gonadotropin-releasing hormone; Gn, gonadotropin.

In bold: The values associated with GH.

*indicates statistical significances of p < 0.05.

In the four POSEIDON groups, the GH-treated cycles showed significantly higher chemical pregnancy rates (group 1: 57.94% *vs*. 26.60%, *p* < 0.001; group 3: 56.82% *vs*. 19.35%, *p* = 0.001; group 4: 46.30% *vs*. 12.24%, *p* < 0.001), clinical pregnancy rates (group 1: 54.21% *vs*. 7.45%, *p* < 0.001; group 2: 35.00% *vs*. 10.00%, *p* = 0.002; group 3: 47.73% *vs*. 9.68%, *p* = 0.001; group 4: 37.04% *vs*. 4.08%, *p* < 0.001), and live birth rates (group 1: 47.66% *vs*. 0, *p* < 0.001; group 2: 28.33% *vs*. 0, *p* < 0.001; group 3: 45.45% *vs*. 0, *p* < 0.001; group 4: 24.07% *vs*. 0, *p* < 0.001) as compared to the non-GH cycles, except the chemical pregnancy rate in the POSEIDON group 2. In addition, the clinical pregnancy loss rates were significantly lower within the GH-treated cycles (group 1: 12.07% *vs*. 100.00%, *p* < 0.001; group 2: 19.05% *vs*. 100.00%, *p* = 0.002; group 3: 4.76% *vs*. 100.00%, *p* = 0.002; group 4: 35.00% *vs*. 100.00%, *p* = 0.049). According to the GEE analysis of several factors, GH supplementation was the significant predictor of chemical pregnancy (group 1: OR 4.18, 95% CI 2.26–7.73, *p* < 0.001; group 3: OR 8.11, 95% CI 2.54–25.95, *p* < 0.001; group 4: OR 5.91, 95% CI 1.87–18.73, *p* = 0.003; **Table 7**) and clinical pregnancy (group 1: OR 19.16, 95% CI 7.87–46.63, *p* < 0.001; group 2: OR 7.44, 95% CI 1.65–33.55, *p* = 0.009; group 3: OR 10.19, 95% CI 2.39–43.52, *p* = 0.002; group 4: OR 27.63, 95% CI 4.46–171.11, *p* < 0.001; **Table 8**) of patients included in POSEIDON groups, except the chemical pregnancy of patients in POSEIDON group 2.

**Table 7:** GEE regression analysis for chemical pregnancy.

Independent variable	POSEIDON group 1			POSEIDON group 2			POSEIDON group 3			POSEIDON group 4		
	OR	95% CI	*p*-value	OR	95% CI	*p*-value	OR	95% CI	*p*-value	OR	95% CI	*p*-value
GH supplementation												
Non-GH	_	_		_	_		_	_		_	_	
GH	4.18	2.26–7.73	<0.001*	1.66	0.56–4.94	0.361	8.11	2.54–25.95	<0.001*	5.91	1.87–18.73	0.003*
Age	0.93	0.81–1.07	0.284	0.95	0.83–1.09	0.437	1.08	0.86–1.35	0.506	1.05	0.89–1.23	0.549
BMI	0.99	0.89–1.10	0.843	1.02	0.90–1.17	0.713	1.23	1.01–1.50	0.043*	1.03	0.84–1.25	0.796
AMH	1.12	0.93–1.35	0.248	0.94	0.74–1.19	0.610	4.00	0.35–45.09	0.262	0.54	0.09–3.44	0.517
FSH	0.92	0.75–1.13	0.430	1.10	0.90–1.36	0.350	0.93	0.72–1.19	0.545	0.79	0.63–1.00	0.048*
AFC	0.91	0.84–1.00	0.049*	1.16	1.04–1.30	0.010*	1.07	0.82–1.39	0.616	1.08	0.86–1.35	0.514
COH protocol												
GnRH antagonist	_	_		_	_		_	_		_	_	
GnRH agonist long	1.82	0.85–3.91	0.125	1.25	0.42–3.78	0.687	2.31	0.53–9.97	0.263	1.43	0.32–6.39	0.640
GnRH agonist ultra-long	2.04	0.66–6.28	0.215	1.05	0.22–5.02	0.956	0.57	0.10–3.16	0.518	0.55	0.11–2.70	0.463
GnRH agonist short	2.02	0.80–5.10	0.137	0.76	0.22–2.68	0.669	1.18	0.25–5.65	0.832	0.71	0.20–2.55	0.601
Duration of Gn	1.14	0.94–1.39	0.182	0.78	0.56–1.08	0.134	0.88	0.59–1.30	0.513	1.16	0.81–1.64	0.417
Gn dose (per 100 IU)	1.00	0.95–1.05	0.931	1.07	1.00–1.15	0.055	1.06	0.99–1.13	0.103	1.00	1.00–1.00	0.837

GH, growth hormone; BMI, body mass index; AMH, anti-Müllerian hormone; FSH, follicle-stimulating hormone; AFC, antral follicle count; COH, controlled ovarian hyperstimulation; GnRH, gonadotropin-releasing hormone; Gn, gonadotropin.

In bold: The values associated with GH.

*indicates statistical significances of p < 0.05.

**Table 8:** GEE regression analysis for clinical pregnancy.

Independent variable	POSEIDON group 1			POSEIDON group 2			POSEIDON group 3			POSEIDON group 4		
	OR	95% CI	*p*-value	OR	95% CI	*p*-value	OR	95% CI	*p*-value	OR	95% CI	*p*-value
GH supplementation												
Non-GH	_	_		_	_		_	_		_	_	
GH	19.16	7.87–46.63	<0.001*	7.44	1.65–33.55	0.009*	10.19	2.39–43.52	0.002*	27.63	4.46–171.11	<0.001*
Age	0.91	0.78–1.08	0.285	0.99	0.84–1.15	0.866	1.02	0.82–1.28	0.853	0.92	0.75–1.13	0.422
BMI	0.94	0.84–1.06	0.346	1.02	0.88–1.18	0.800	1.24	1.03–1.50	0.027*	1.07	0.83–1.37	0.598
AMH	1.06	0.85–1.32	0.594	0.92	0.71–1.19	0.521	2.06	0.19–22.37	0.552	0.37	0.04–3.79	0.399
FSH	0.86	0.67–1.11	0.254	1.13	0.89–1.43	0.308	0.91	0.70–1.18	0.456	0.66	0.44–0.97	0.034*
AFC	0.89	0.80–0.99	0.027*	1.18	1.04–1.34	0.009*	1.06	0.82–1.37	0.676	0.94	0.70–1.27	0.700
COH protocol												
GnRH antagonist	_	_		_	_		_	_		_	_	
GnRH agonist long	1.52	0.62–3.73	0.359	0.61	0.18–2.02	0.418	1.30	0.27–6.29	0.748	1.80	0.31–10.38	0.508
GnRH agonist ultra-long	1.53	0.44–5.31	0.506	0.43	0.08–2.40	0.338	0.51	0.08–3.22	0.470	0.36	0.05–2.66	0.318
GnRH agonist short	1.51	0.49–4.70	0.472	0.77	0.18–3.36	0.732	0.82	0.14–4.95	0.832	1.50	0.30–7.44	0.621
Duration of Gn	1.25	0.98–1.58	0.071	0.95	0.66–1.37	0.788	0.90	0.59–1.38	0.629	1.63	1.03–2.57	0.036*
Gn dose (per 100 IU)	0.98	0.92–1.04	0.522	1.01	0.93–1.09	0.863	1.06	0.99–1.15	0.112	1.00	1.00–1.00	0.491

GH, growth hormone; BMI, body mass index; AMH, anti-Müllerian hormone; FSH, follicle-stimulating hormone; AFC, antral follicle count; COH, controlled ovarian hyperstimulation; GnRH, gonadotropin-releasing hormone; Gn, gonadotropin.

In bold: The values associated with GH.

*indicates statistical significances of p < 0.05.

## Discussion

This study explored the role GH played on the IVF/ICSI outcomes for POR patients in each POSEIDON subgroup who failed an IVF/ICSI cycle followed by a subsequent GH-prescribed cycle. Our findings revealed that GH supplementation could enhance the number of embryos available to transfer and improve the clinical pregnancy rate as well as the live birth rate for all low-prognosis patients who failed a previous IVF/ICSI cycle. GH could also significantly enhance the number of oocytes retrieved in POR subgroups with normal ovarian reserve and increase the number of day-3 good-quality embryos in subgroups either with normal ovarian reserve or aged young.

Regarding live birth, women in all four POR subgroups presented with optimistic results and ended up with the live birth rate to be 47.66%, 28.33%, 45.45%, and 24.07% in POSEIDON groups 1, 2, 3, and 4, respectively. The clinical pregnancy rates were significantly increased in the subsequent GH-addition cycles among all the four POSEIDON groups as well. These findings signified the feasibility in adding GH to women with POR who experienced a failed IVF/ICSI cycle. The systematic review and meta-analysis by Cozzolino et al. (15), which included 12 randomized controlled trials (RCTs) with 1,139 patients diagnosed as poor responders according to different criteria, found that GH adjuvant therapy could help to improve the clinical pregnancy rate while not being able make a difference in the live birth rate. However, another recent meta-analysis including 15 RCTs with 1,448 patients characterized as poor responders under variable criteria demonstrated that both the clinical pregnancy rate and the live birth rate enhance significantly with GH supplementation (21). This controversy might be attributed to the discrepancy of the POR definitions and the heterogeneity in the POR population included in the previous studies. In our study, each subgroup was supposed to be homogeneous based on the POSEIDON criteria. The improved clinical pregnancy rate and live birth rate indicated that for low-prognosis women who failed a previous IVF/ICSI cycle, regardless of their age and ovarian reserve, GH supplementation might be a potential choice to improve the pregnancy outcome.

The ameliorative pregnancy outcome might be due to the elevated number of embryos available to transfer. Our results manifested that GH could raise the number of embryos available for transfer in all the four subgroups of women with POR. In several previous RCTs which applied GH as an adjuvant therapy in IVF/ICSI cycles in women with POR under Bologna criteria, the numbers of transferred embryos were significantly enhanced (22–24). In the review by Cozzolino et al. (15) including poor responders according to different definitions, the number of embryos available to transfer was also significantly increased. Our findings, along with all these previous results, confirmed that the number of transferrable embryos was probably raised after the addition of GH for women with POR, whichever subgroup they were in.

In previous studies among patients with POR diagnosed by the Bologna criteria, the number of oocytes retrieved was significantly increased after the addition of GH (22–24). In this study, by stratifying POR patients into specific subgroups according to the POSEIDON criteria, we identified that not all POR patients but only those with normal ovarian reserve (POSEIDON groups 1 and 2) exhibited a significantly elevated number of oocytes retrieved. A retrospective study enrolling POR patients with impaired ovarian reserve in POSEIDON groups 3 and 4 manifested that the numbers of oocytes retrieved were comparable in both groups (25). This result was to some perspective consistent with ours, which also indicated that the beneficial effect on the retrieved oocyte number by GH adjuvant was mainly among POR patients with normal ovarian reserve instead of those with poor ovarian reserve.

The growth hormone receptor (GHR) expressed widely on granulosa, theca, and luteal cells in the ovary (15). GH supplementation was reported to be able to increase the receptor density for the FSH receptor (FSHR), LH receptor (LHR), and GHR in the granular cells (13) and might improve follicular FSH responsiveness, which could contribute to the oocyte number retrieved for the poor responders. Moreover, GH was reported to promote the *in vitro* maturation of human germinal vesicle (GV) oocytes (26), which might also lead to raised oocyte number. POR patients with normal ovarian reserve were supposed to have a larger follicular pool with the potential to provide more mature oocytes to be retrieved. Our results put forward an opinion that POR patients with normal ovarian reserve, regardless of their ages, might benefit more from GH supplementation during the IVF/ICSI cycles.

In the meanwhile, we discovered that the numbers of day-3 good-quality embryos increased not only in the POR subgroups with normal ovarian reserve but also in POR patients with impaired ovarian reserve yet aged young (POSEIDON group 3). Our results might imply that young women with POR could benefit from GH in gaining good-quality oocytes. In a study including cumulus–oocyte complexes (COCs) collected from women aged 26 to 46 years, the authors exhibited that among POR patients ≤ 34 years, the proportion of the good-quality oocytes (grade 2.5 and 2) was elevated with GH supplementation, while among POR patients aged above 35 years the proportion of grade 2.5 oocytes was still increased yet grade 2 oocytes decreased (8). This implicated that GH adjuvant might contribute more to the young POR patients in terms of improving the overall good-quality proportion. It uncovered that the effect of GH on quality of oocytes and embryos among young POR patients needs more attention and further studies with larger sample size.

The strength of this study was to define women with POR under POSEIDON criteria and to stratify them into groups with homogeneous clinical characteristics. Additionally, we applied a longitudinal design and compared between the two IVF/ICSI cycles performed within 12 months for the same patients, which helped to control the confounders. The limitation lay on the retrospective nature of the study and the restriction of a previous failed IVF/ICSI cycle for our participants, which narrowed the application range of patients in the conclusion.

## Conclusion

In conclusion, growth hormone supplementation might improve the clinical pregnancy rate and the live birth rate for low-prognosis women diagnosed by POSEIDON criteria who failed a previous IVF/ICSI cycle. The application of growth hormone for low-prognosis women who failed IVF/ICSI cycles previously might be a feasible choice and needs further research.

## Data Availability Statement

The raw data supporting the conclusions of this article will be made available by the authors, without undue reservation.

## Ethics Statement

The studies involving human participants were reviewed and approved by the Institutional Review Board (IRB) of the Center for Reproductive Medicine, Shandong University. The patients/participants provided their written informed consent to participate in this study.

## Author Contributions

XJ and PL designed the study. XL, JX, and LB collected data and conducted data analysis. PL and XL drafted the manuscript. XJ revised the manuscript critically. All authors contributed to the article and approved the submitted version.

## Funding

This study was funded by the National Key Research & Developmental Program of China (2018YFC1003803), Natural Science Foundation of Shandong Province (ZR2019PH009), the National Science Fund for Distinguished Young Scholars (82125014), the National Natural Science Foundation of China (82071609, 81771541, 82171651, 81971352), Taishan Scholars Program for Young Experts of Shandong Province (tsqn20161069), and the Young Scholars Program of Shandong University.

## Conflict of Interest

The authors declare that the research was conducted in the absence of any commercial or financial relationships that could be construed as a potential conflict of interest.

## Publisher’s Note

All claims expressed in this article are solely those of the authors and do not necessarily represent those of their affiliated organizations, or those of the publisher, the editors and the reviewers. Any product that may be evaluated in this article, or claim that may be made by its manufacturer, is not guaranteed or endorsed by the publisher.
